# Studying Peptide-Metal Ion Complex Structures by Solution-State NMR

**DOI:** 10.3390/ijms232415957

**Published:** 2022-12-15

**Authors:** Deborah E. Shalev

**Affiliations:** 1 The Department of Pharmaceutical Engineering, Azrieli College of Engineering Jerusalem, 26 Yaakov Shreibom Street, Jerusalem 9371207, Israel; debbie@jce.ac.il; Tel.: +972-2-6591830; 2 The Wolfson Centre for Applied Structural Biology, The Hebrew University of Jerusalem, Edmond J. Safra Campus, Jerusalem 9190401, Israel

**Keywords:** peptides, metallopeptides, peptide chelates, peptide structure, NMR, structural NMR

## Abstract

Metal chelation can provide structural stability and form reactive centers in metalloproteins. Approximately one third of known protein structures are metalloproteins, and metal binding, or the lack thereof, is often implicated in disease, making it necessary to be able to study these systems in detail. Peptide-metal complexes are both present in nature and can provide a means to focus on the binding region of a protein and control experimental variables to a high degree. Structural studies of peptide complexes with metal ions by nuclear magnetic resonance (NMR) were surveyed for all the essential metal complexes and many non-essential metal complexes. The various methods used to study each metal ion are presented together with examples of recent research. Many of these metal systems have been individually reviewed and this current overview of NMR studies of metallopeptide complexes aims to provide a basis for inspiration from structural studies and methodology applied in the field.

## 1. Introduction

Ten of the twenty elements that are considered essential for life are metals. These include Na, K, Mg, Ca, Fe, Mn, Co, Cu, Zn and Mo [1]. The ability to probe metal-bound proteins and peptides is essential for understanding the biological role of metals. Non-essential metals also bind in the biological context—some lead to toxicity and some are therapeutic. Solution state nuclear magnetic resonance (NMR) is commonly used to elucidate protein structures in environments that emulate the biological milieu to the best of our ability [2]. NMR is used to study metal ion-protein complexes [3], however, these systems can be challenging to solve by NMR or by X-ray crystallography. Elucidating binding between protein residues and metal ions does not necessarily require solving the entire protein complex: Peptides derived from a binding site of interest are often used to provide information about the mechanism and binding mode of a metal ion ligand to a protein binding site. Bioactive peptides also bind metals, and metal-binding can stabilize or catalyze processes involving peptides. The disadvantage of using peptides revolves around their inability to maintain a rigid, stable conformation in solution, and our inability to ascertain that the amino acids in the peptide are indeed in the same configuration as would be found in the binding site of the protein. Nonetheless, peptides can serve as enticing models for studying metal-bound complexes and are continuously used to determine both mechanism of binding and binding modes. Metallopeptide complexes provide vastly simplified systems that are useful for specifically studying regions of binding and are more amenable to many analytical methods including NMR. Many naturally occurring peptides are also studied by NMR, particularly as their conformational flexibility usually hinders the crystallization step necessary for X-ray crystallography structure determination. This paper reviews recent NMR structural studies of peptide-metal complexes with the essential metals and other biologically relevant metals.

## 2. Metal Binding in Biological Systems

Metal binding is ubiquitous in biological systems as are the metals themselves. A recent statistic from the Metal PDB [1] shows over 30% of the protein structures in the Protein Data Bank [2] bind metal. Metal ions are often at the core of structural stabilization [3] and they can be essential for biological functionality [4] including catalysis [5] and inhibition or acceleration of aggregation [6]. Metal homeostasis is regulated by the metal-binding metallothioneins that also protect against heavy metal toxicity [7].

Whereas van der Waals interactions, hydrogen bonds, hydrophobic interactions and salt bridges within a protein provide tertiary stability, there are cases where non-contiguous regions require even further stabilization. This can be provided by covalent disulfide bonds between cysteine residues or by way of complexation with metals. Most notable of these are iron, copper, zinc and calcium [8]. Furthermore, metal ions can govern protein dimerization and direct homo- or heterodimerization [9]. Metal ions serve as cofactors in catalytic sites of two thirds of the known biocatalysts, where they are required for proper function [10,11]. Metal-binding can also inhibit bioactivity, as in the case of zinc(II) and copper(II) ions that can inhibit fungal and bacterial growth [12].

Metal-binding irregularities play a role in diseases such as Alzheimer’s [13,14], ALS [15] and neurodegenerative disorders [6], among many others. Nickel and the essential metal, cobalt, are among the metals that can cause allergic reactions in humans [16]. Both excess and deficient concentrations of metal ions in the body can cause mild to fatal diseases. For example, Wilsons disease, that results in a buildup of copper in the body [17] can be treated by copper-chelation therapy [18]; whereas copper deficiency due to Menkes disease [19], which causes copper deficiency in cells, is usually fatal, although copper therapy has been shown to ameliorate the condition [20]. Likewise an excess of iron, as in that caused by the genetic hemochromatosis [21], is treated using iron-binding sorbents [22]; and iron deficiency, anemia, is treated by iron supplements or transfusion [23].

Analyses of protein-metal complexes by NMR are always challenging because the position of complexation within the protein needs to be determined by other means, however their prevalence in nature requires their study and many excellent reviews have been written on the subject [24,25,26,27]. In cases of paramagnetic metal ions, the region around the iron is effectively erased from the NMR spectrum due to paramagnetic line-broadening from fast relaxation. The main paramagnetic effects in solution-structure NMR are paramagnetic relaxation enhancement (PRE), pseudo-contact shifts (PCS) and residual dipolar couplings (RDC). Recent methodologies have enabled studying paramagnetic metal protein complexes by exploiting these properties and taking advantage of fast relaxation that reduces acquisition times, and the long-range interactions that can provide distance information of up to 35 Å [28,29,30,31]. These have extended the range of targets available for structural analysis including drug discovery [32] and the study of metalloproteins [31].

## 3. Using Peptides as Biomimetics for Metal Binding

The prevalence of metal ion-binding proteins invites the design of peptides as proteinomimetics [33] together with directly studying native metal-binding peptides. It is difficult to study the structures of peptides due to their inherent flexibility that often defies attempts at crystallization and challenges structure determination by solution-state NMR due to large conformational diversity. Despite the difficulties in using peptides in structural studies, they are commonly used as protein-mimetics for metal-binding of essential [34] and potentially toxic metals [35] and in drug design.

## 4. Applications of Metal-Binding Peptides

Protein sequences, structures and binding sites have been optimized by nature over millennia. The amino acid building blocks are both chemically and structurally versatile and also bind other biological macromolecules efficiently and specifically. Many peptides occur naturally or are cleaved from parent proteins. Therefore, natural, derived, and synthetic peptides are ideally suited for many purposes and occur in a myriad of applications [36]. Therapeutic peptides are becoming more prevalent [37] and include anticancer [38], antimicrobial [39], antiviral [40], and anti-neurodegenerative diseases [41], among others. Peptides can self-assemble into structures with properties including nanostructures [42] and microstructures [43] for injectable hydrogels [44], tumor treatment [45], and drug delivery [46].

Protein-ligand interactions can be mimicked using peptides derived from the binding region of the relevant protein [47]. These peptides take advantage of the evolutional honing of the protein sequence and the innate ability of peptides to chelate metals. The peptides can comprise the specific binding residues from the protein binding site, but are also easily modified using other amino acids, noncanonical amino acids and modifications to the backbone, termini and by cyclization [48]. These modifications confer conformational stability onto the peptides, and also increase metabolic and chemical stability which all increase the range of applications that can be addressed using peptides.

Peptides are found throughout the industrial world. Novel therapeutics can be formed using metallopeptides: Certain antimicrobial peptides naturally feature an amino terminal copper and nickel-binding (ATCUN) motif [49,50], and the specificity, solubility and stability of other antimicrobial peptides have been improved through metal-binding to increase microbial resistance [51,52]. Peptides with antitumor activity include, e.g., cytotoxic gold-peptide complexes [53] and bicyclic peptides developed to bind metal for potential radiopharmaceuticals [54]. Peptides that feature metal-binding sites can function as biocatalysts [55] and can also be used to direct chiral synthesis [56].

Metal ions can mediate self-assembly of peptides to create supra-molecular structures, including those which can function as catalysts [57,58] or assemblies with modifiable nanocavities with the potential for enantioselective recognition [59].

Peptides are used as recognition elements in molecular probes due to their specificity in binding [60,61], and their ability to bind metals can be used to detect the metals themselves [62,63]. Metallopeptides are also used as electrical probes for detecting bacteria [64,65].

Another aspect of metal-peptide complexes is the chemical stabilization of the metal itself due to chelation. In the food industry, peptides are used as chelators to increase bioavailability of mineral supplements by chemically protecting them from oxidation and by changing their solubility properties [66,67].

The ability of peptides to bind metals was also used to improve the biocompatibility of gold nanoclusters for imaging [68] and bound lanthanide was used as an imaging agent [69]. Metal remediation can also be achieved using the specific binding between natural peptides and heavy metals [70,71].

## 5. Challenges of Using Peptides

Peptides are nonetheless challenging to use: They are chemically unstable [72] and are prone to undergo hydrolysis at the peptide bond, oxidation [73], aggregation [74], internal cyclization [75], deamidation [76] and to form diketopiperazine and pyroglutamic acid [77]. Peptides can be enzymatically hydrolyzed by peptidases and therefore lack metabolic stability [78].

Peptides that are derived from metal-binding sites in proteins may bind the ligand with lower affinity since not all the binding moieties are necessarily preserved in the peptide and their geometry may not have the precise orientation that matches the coordination geometry of the metal ion due to backbone flexibility. The lack of conformational stability often results in diminished specificity and reduced binding capabilities necessitating some type of conformational tethering or stabilization. This can be done via cyclization using metals, amides, disulfide bridges and backbone methodology [48].

It is challenging to produce peptides at scaled-up quantities although new methodology is significantly improving yields [79,80,81,82].

## 6. Use of NMR in the Study of Peptide-Metal Complexation

The inherent flexibility of peptides renders them unlikely to crystallize for use in X-ray crystallography studies. This leaves NMR as the method of choice, and peptide-metal complexes have been studied extensively by NMR as will be described below.

There are many ways to classify metals in biological systems, but from a structural NMR point of view, metals can be categorized into diamagnetic and paramagnetic metals [83] according to their effect on the acquired spectra. Diamagnetic structures of peptide complexes are easily obtained using standard structural methodology that usually involves through-bond experiments to determine the identity of the amino acids, and through-space and dipolar interactions to both determine the sequential identity and to provide distance restraints for structure calculations [84]. Peptides and proteins complexed with paramagnetic ions suffer from broadening due to enhanced transverse relaxation [30] at distances of up to many angstroms from the paramagnetic center, thereby creating a blind sphere around the metal ion. In peptide-metal systems this region may encompass the entire complex due to their small size—effectively erasing the entire NMR spectrum. This precludes using canonical methods for solving the structures of paramagnetic complexes. Line-broadening can be so detrimental to the spectra that in many cases paramagnetic ions are removed from samples prior to NMR analysis [85]. None-the-less, since the degree of peak broadening is proportional to the distance from the paramagnetic ion, a paramagnetic ion can also provide information that can be used for structure determination, especially if the paramagnetic complex can be compared to a corresponding diamagnetic complex [28,86].

Chemical shift mapping is used to identify the binding site and is done by titrating the apo peptide with the metal ion and following perturbations in chemical shift and line-broadening in the NMR spectrum to determine the position of interaction of the ion on the peptide [27]. The coordination geometry of metal ions also requires experimental determination as the mode of binding and ligands can alter the bound structure [27].

The inherent flexibility of linear peptides can be addressed through cyclization that limits the conformational freedom of the peptide and increases bioavailability of the complex [87]. Cyclization can be done in many manners and the size and orientation of the binding functional groups can be tuned to match the metal coordination spheres, which can significantly increase the binding coefficient [88]. Many studies of bound and apo cyclic peptides have been done and are discussed in the following sections, and the process of the transition between the apo and bound states can also be addressed as in the case of a cyclized analog of the ATCUN motif [89].

For the purposes of this review, a “peptide” will be defined as a molecule with less than 23 residues since this is the minimal sized polypeptide that the Protein Data Bank (https://www.rcsb.org/ accessed on 13 November 2022) will accept, although longer polypeptides will be addressed as well. The screen for recent studies on the individual metal-peptide complexes was done on all the essential metals for the period spanning 2012–2022 using the Clarivate Web of Science search engine (https://www.webofscience.com/ accessed on 13 November 2022) and search terms NMR, structure* OR structural, binding, peptide* and each relevant metal in the Topics field. Common non-essential metals are addressed as well. Only solution NMR studies were considered. The metals are listed according to atomic number.

## 7. Diamagnetic Metal-Peptide NMR Studies

Diamagnetic metal-peptide complexes can be studied directly by common 2D NMR methodology [84,90]. In some cases the naturally abundant metal isotope is NMR-active which enables determining stoichiometry and binding coefficients directly [91]. The challenge is to determine the correct binding ligands since, in most cases, these are via heteroatom lone pairs and are not directly detectable by NMR. Although the peptide structure can be determined to high resolution, part of the structure determination process is to define the bond-lengths and geometries between the peptide and metal-ion ligand. Since peptides are so flexible, they can easily conform to incorrect geometries during the structure calculation step, resulting in erroneous structural results. The correct binding geometry and ligands must be determined prior to structure determination. The binding mode can be taken from crystallographic structures, or methods for predicting metal-binding sites in proteins (e.g., [92,93,94]), but even here, one must consider that experimental conditions in the solution sample differ from crystallization conditions and can potentially change the in vitro mode of binding. This may result in an experimental error if not detected, or it can provide additional information regarding binding modes under varied conditions, which is a useful application for peptide studies (e.g., [95]).

### 7.1. Sodium and Potassium

Although sodium and potassium ions are both essential and ubiquitous, they do not bind sufficiently specifically or with high enough affinity in the solution state to create complexes that are amenable to NMR.

### 7.2. Magnesium

Magnesium is both an essential and abundant element that is present in the body as Mg(II). Many proteins bind Ca(II) and Mg(II), though the latter is smaller which changes its binding affinity [96]. Mg(II) binds proteins weakly with an octahedral geometry [97,98]. The weak binding in proteins suggests that binding with peptides will be prohibitively weak, and correspondingly no studies have been performed on magnesium-peptide complexes within the length defined. Larger peptides have been studied, e.g., the 76 amino acid N-terminal domain of calmodulin, to understand the difference in binding between Ca(II) and Mg(II) [99]: This structure was solved with the use of residual dipolar coupling (RDC) [100] methodology due to the flexibility of the polypeptide [101].

### 7.3. Calcium

Calcium is another essential and plentiful element that participates in cellular signaling in its ionic form, Ca(II) [102]. Binding to Ca(II) can be identified via chemical shift mapping of proton or heteroatom signals, as in the case of a six-amino acid peptide derived from the repetitive sequence in silk-moth silk. This peptide showed significant changes in 1D ^13^C chemical shifts upon binding Ca(II), which was measured directly in solution. Whereas this only gave an indication of binding, it is a strong tool for identifying binding where necessary [103].

One of the more studied primary calcium binding proteins is calmodulin [104]. In its Ca(II)-bound form, calmodulin binds to calcium channel proteins and blocks them. Although it is often referred to as a peptide, it is about 150 amino acids in length requiring 3D NMR methodology [90], often together with RDC refinement, to obtain structures for the calcium-bound peptides. Calmodulin has four calcium-binding loops, where the N- and C-terminal derived peptides bind calcium with different affinities and are often studied separately [105,106,107]. The coordination geometry of Ca(II) in EF-hand proteins has been established as a pentagonal bipyramid and coordinates oxygen atoms [108]. Paramagnetic probes can be introduced into the molecule by performing two experiments in which the Ca(II) ion is exchanged, respectively by different lanthanides that have dissimilar magnetic susceptibility anisotropy tensors, such as Tb(III) or Tm(III): Internuclear vectors referenced to each lanthanide provide additional information regarding internal orientation relative to the paramagnetic probe [109,110]. The relative dynamic motion of the bound and apo structures of calmodulin can be measured by ^1^H-^15^N transverse relaxation optimized spectroscopy-heteronuclear single quantum coherence (TROSY-HSQC) experiments [111].

### 7.4. Copper (I)

Copper is an essential metal and plays an important biological role both in structure and biochemical reactions. Copper-transport is done by copper chaperones due to its toxicity. This is especially due to its ability to switch between its reduced, Cu(I), or oxidized, Cu(II), states [112]. From the point of view of structural NMR, these two states are particularly different because Cu(I) is diamagnetic and Cu(II) is paramagnetic. A main challenge with working with Cu(I) is that samples require careful preparation in a glove box and the sample itself must be well-sealed to prevent oxidation by air [113,114]. Following this, structures can be determined directly, and Cu(I) complexes with peptides can be used to mimic copper-binding proteins and chaperones.

Linear and cyclic peptides have been used as models for the conserved sequence of binding sites. The binding modes were investigated both by mutation studies and by changing the environmental conditions., e.g., mutations of the preserved Met residue in the conserved sequence of copper chaperones severely inhibited copper complexation [115] and changing the pH of the environment resulted in different binding modes [95,116]. These suggest that pH conditions may change the coordination sphere of the metal, showing how peptide-metal models can help elucidate possible release mechanisms. A structural study on the interaction of Cu(I) with the N-terminal Aβ16 fragment of amyloid beta (Aβ) was performed to determine whether all three His residues participated in metal ion binding [117]. Both these studies were done on small peptides derived from the binding site of larger molecules. Since the binding mode was not clear, these studies took the experimentally derived dipolar interactions (nOe restraints) and applied them to the peptides and did the minimization calculations while introducing different plausible metal-binding modes. The resulting ensembles were analyzed and the one that best fit the experimental data, i.e., lower RMSD, where all experimentally derived distance constraints were upheld, was assumed to represent the structure of the complex.

### 7.5. Zinc

Zinc is among the main ions for structural, catalytic, and regulatory functions in proteins and is found in an estimated 10% of all proteins [118,119]. Of these, the most prevalent are the zinc finger proteins that are representative of flexible proteins that are stabilized via metal-binding. Short and longer peptides have been used extensively for modeling and understanding different aspects of zinc finger binding, including metal coordination, folding and actual binding [120,121,122]: Cyclic peptides with linear tails showed conformational and thermodynamic stability relative to linear peptides for modeling zinc fingers, and were able to fold into conformations that reproduced the zinc-ribbon fold of zinc fingers and bound Zn(II) better than their linear counterparts [123]. Peptides were used to study secondary structural elements common in zinc fingers and their role in folding and binding zinc: This enabled designing peptides that were modified by 23% of their native residues and still showed tertiary folds and stability on par with the natural backbone of the original zinc finger protein [124].

Zinc-binding can cause significant conformational change in peptides [125] and has been shown to induce oligomerization of amyloid beta, a 42 amino acid polypeptide. Amyloid beta is particularly difficult to study structurally because it forms aggregates spontaneously. Therefore, these processes are best conducted using truncated peptides that preserve the beta-sheet formation region but that do not easily undergo complete oligomerization themselves. For example, peptides were used to study the familial Taiwanese mutation D7H region of amyloid beta that affects zinc-induced oligomerization by forming a stable homodimer via zinc-binding. 

Rats are resistant to Alzheimer’s disease: The zinc-induced dimerization in rat amyloid beta protein was also studied using a 1–16 truncated peptide and showed that the C-termini of the two peptides dimerized such that they were positioned in opposite directions from each other, which prevented further aggregation [126,127]. Elucidating this interface may help to rationally design drug compounds to block the plaque-forming processes.

Bioinspired zinc-bound dipeptides have shown self-assembly into stable nano-superstructures [128] with enzymatic [129], fluorescent [130] and electromechanical properties [131]. Chemical shift perturbations were studied by NMR to predict the coordination structure [130].

Zinc participates in additional binding events such as within metal chaperones where zinc-binding was modelled using a small cyclic peptide derived from the conserved binding residues [95], and postulated zinc-binding motifs in disulfide-rich peptides [132].

### 7.6. Molybdenum

Molybdenum is the only 5th row element among the essential metals and is only biologically active upon complexation to form a molybdenum cofactor [133]. Deficiency in the molybdenum cofactor results in a fatal metabolic disorder [134]. There are no studies of molybdenum-peptide systems by NMR, however there are studies on molybdenum-peptide systems in general, to which NMR could feasibly be applied. Briefly, these include known molybdenum-peptide complexes such as those in Noni juice, which is used medicinally [135]; an antimicrobial assembly comprising molybdenum-polyoxometalate and a positively charged peptide [136]; MoS_2_-bound peptide complexes have been studied [137], including materials that disrupt the structure of amyloid fibrils [138] and are used as electrochemical sensors [139], e.g., as potential anticancer and antibacterial agents [140,141,142]. Furthermore, tungsten is becoming more common in our environment and may inhibit molybdoenzymes through exchange with the bound molybdenum ion in the cofactor [143], making this an increasingly relevant system to study.

## 8. Paramagnetic Metal-Peptide NMR Studies

New tools and methodology take advantage of the fast relaxation induced by paramagnetic centers to reduce overall experiment duration through shortened acquisition times and recycle delays [29,83]. The transition metals differ in their pseudo-contact shifts (PCSs), evident in significant changes in chemical shift upon binding, and their paramagnetic relaxation enhancement (PRE), which reduces or eliminates NMR signals due to broadening out [144]. The degrees of PCS and PRE determine the ability to use a given ion to identify the binding species by way of PCS or to identify binding residues with chemical shift mapping due to PRE.

Smaller systems are more challenging because the range over which NMR signals are erased can encompass the entire complex, but new approaches have been shown to reduce the blind sphere around the paramagnetic ion, enabling studies on small protein systems, such as the high potential iron-sulfur protein, PioC, with its [Fe_2_S_4_]^2+^ cluster [145], and a study of exchange rates of free and copper(II)-bound complexes [146].

Many of the analyses conducted on paramagnetic complexes did not include structure determination. However, 1D NMR was used extensively to follow line-broadening as a function of titration, where the degree of broadening correlates the proximity to the paramagnetic element, and changes in chemical shift indicate a binding event or structural changes upon binding.

In some cases, experimental structures were determined directly on samples with low metal-to-peptide molar ratios: The peptide is assumed to be non-structured in its unbound form and to be in equilibrium with a more rigid structure adopted upon binding. The dipolar interactions from the unbound fraction are considered to be negligible, whereas those arising from proximate hydrogen atoms in the bound form are used to calculate the bound structure. Subsequently, canonical methodology is used to calculate a representation of the bound structure from empirical nOe interactions.

The precise chelation mode of the metal ion within the bound structure requires additional experimentation. Titrating peptides with the ion and following signal broadening and changes in chemical shift as a function of titration can provide necessary information regarding the positioning of the metal ion, assuming that the signals emanating from hydrogens that are proximate to the paramagnetic binding center are those that show the most significant change. Careful titration can also indicate the order with which the peptide moieties complex the metal ion.

In the following studies, structural data was generated by determining the structure of the peptide by various modelling methods, and then assigning the NMR spectra and identifying the binding sites by following line-broadening using 1D NMR. Where given, the proposed metal-bound structures were generated by positioning the metal ion in the experimentally determined binding site using UCSF Chimera [147].

### 8.1. Manganese

Manganese is an essential element and plays a role in enzyme activity and metabolic regulation. It binds magnesium superoxide dismutase, which is responsible for scavenging reactive oxygen species. Excess or deficiency in manganese can lead to adverse health effects [148].

Destabilization and misfolding caused by manganese-binding in prion disease [149] was studied with a 30-amino acid peptide comprising three repeats of a decapeptide repeat in the C-terminal region of the calcium protein, Cap43, that was probed using 1D ^1^H-NMR to follow line-broadening upon binding and elucidate the role of divalent ions in the pathogenesis of prion disease [150]. Mn(II) ion binding to a peptide derived from amyloid beta, Aβ(13–23), was also studied by measuring line-broadening upon binding by 1D ^1^H-NMR [151].

Mn(II)-binding with a 30-amino acid peptide derived from the protein YPk9, a protein that may protect against manganese toxicity in Parkinson’s disease, was modeled based on the chemical shift data obtained by NMR [152].

### 8.2. Iron

Iron is an obvious essential element which participates in oxygen transport in the body. Both surplus and iron deficiency cause disorders [153,154]. Both ferric and ferrous forms of iron are paramagnetic and the transformation between these forms is central to their activity. The facile oxygenation of ferrous to ferric iron ions requires preparing samples in a glove box in an oxygen-free environment to prevent unwanted reactions. The following studies show creative tactics to study iron, and exemplify the utility of a multidisciplinary approach.

Iron binding is clearly detected via line-broadening of the most proximate amino acids. An example is a study done by grafting a six-residue iron-binding motif onto a 29-residue peptide and using NMR to detect line-broadening as indicative of specific interactions between the peptide and Fe(III). Circular dichroism, isothermal titration calorimetry, capillary zone electrophoresis, thermal denaturation, and computational approaches were used to elucidate the binding mode and structure of the peptide model system [155].

Biomineralization in magnetotactic bacteria is regulated by magnetite-associated proteins that have short sequences that bind iron. Peptides of these iron-binding regions were reacted with Fe(II), Fe(III), Ni(II), and Zn(II) to determine specificity, binding coefficients and binding residues by NMR. Coprecipitation was subsequently used to determine the significance of each of the binding residues [156]. Computational methodology was also combined with NMR to determine structures of artificial peptides that adopt chiral helicate complexes with Fe(II) and Co(II) [157].

1D and 2D NMR methods that can directly detect paramagnetic complexes were demonstrated on the 8-amino acid microperoxidase-8 bound with heme iron, as a model peptide for the cytochrome C binding site. 1D spectra were acquired using excitation sculpting with gradients to suppress the water for the Fe(II)-bound samples and a superWEFT pulse sequence [158] was used to measure the Fe(III)-bound samples [159].

### 8.3. Cobalt

Cobalt is present in the body in minute amounts but is none-the-less an essential element. In cases of excess, cobalt toxicity stems from its ability to produce reactive oxygen species and to substitute iron in metalloenzymes, rendering them inactive [160]. Cobalt is commonly found in its Co(II) or Co(III) forms, where most cobalt forms are paramagnetic. Co(III), however, has high- and low-spin configurations, where the latter is diamagnetic [160]: E.g., A diamagnetic Co(III) complex with a peptide-porphyrin conjugate was solved using standard methodologies [161]. High-spin Co(II) has relatively large PCSs and small PRE making it amenable to structure determination [144].

A model of the structure of a complex of Co(II) and a fragment of amyloid beta protein, Aβ(13–23), was determined by canonical methodology on a sample with a 1:0.01 molar ratio of peptide-to-cobalt. The intensity of the signals were measured as a function of titration and signals with reduced intensity were considered to be proximate to the cobalt ion and served as the cobalt binding center [151]. The binding site of a cobalt(III)-Schiff base complex to another amyloid beta protein fragment was determined using ^1^H-NMR, again, by identifying the binding histidines through line-broadening [162].

Another indication of binding came from an analysis of cobalt(II) binding to fibrinopeptide B, a factor in thrombosis, where the peptide structures were determined in the presence of cobalt(II) and gadolinium(III) [163]. In this case, the metal ions were not shown in the structures.

The previously mentioned 30-amino acid peptide that binds manganese, derived from a decapeptide repeat in the calcium protein, Cap43, was also found to bind Co(II). Titrating the free peptide to give the cobalt complex showed line-broadening as a function of interaction which enabled identifying the binding histidine residues. Furthermore, the pH range at which binding occurred was identified as part of the binding mechanism. Models of the postulated coordination spheres of Co(II) were generated and minimized based on the NMR data and using HyperChem™ [150].

The conformation of peptides bound to chiral cobalt oxide nanoparticles was determined to study chiral evolution: Upon binding, the tripeptide ligands showed a number of sets of peaks due to the high PCS of the nanoparticles that could be used for structure determination due to their low PRE [164].

### 8.4. Copper (II)

Copper(II) is the heaviest of the paramagnetic essential metals. Copper(II) binding has been studied in numerous metal-binding proteins [165,166]. Studies have used signal broadening to identify binding residues [167] and these can also be indicative of the order of binding [168]. When ^1^H spectra broaden out, additional information can be gleaned from the corresponding ^13^C spectra, e.g., [169]. In general, these studies require assigning the ^1^H spectrum and then titrating with Cu(II) to identify the binding groups. Subsequently the bound complex can be determined experimentally [168] or in silico [170] and the Cu(II) ion can be positioned in the determined binding site, resulting in a bound structure.

Due to the difficulties in measuring copper(II)-bound complexes, diamagnetic metals with square planar binding geometry are often used to derive a bound structure. The position of copper binding can subsequently be determined through line-broadening analysis and if there is experimental evidence that the binding mode is the same, Cu(II) can be substituted in the structure calculation and copper(II)-bound structures can be determined. This methodology has been demonstrated with peptide-metal complexes using palladium(II) [171], silver(I) [172], and nickel(II) [173,174].

Line-broadening analysis on ^1^H spectra was used to determine residues that participate in binding Cu(II) to model copper(II)-binding sites [175] and in copper(II)-mediated aggregation [176]. Line-broadening in ^13^C spectra can also be applied [177] and chemical shift deviations can be followed using ^1^H-1D and 2D ^15^N-HSQC ^13^C-HSQC and ^1^H-^1^H-TOCSY experiments. Precise determination of the binding nitrogen in histidine residues was shown using proton spin-lattice relaxation rate studies [178]. Cyclic peptides were designed to bind Cu(II), where binding was determined through line-broadening analysis [179], and to follow Cu(II)-driven aggregation [176].

## 9. Metal Substitutions

In many cases paramagnetic ions can be substituted by diamagnetic ions with the same binding geometry to either model the paramagnetic complex or to provide a peptide structure into which the paramagnetic ion can be inserted for structure generation. The opposite can also be done in cases where the longer-range interactions with the paramagnetic center are desired, in cases where PCS dominates the PRE.

As described above, this was done in the case of the paramagnetic Cu(II) with diamagnetic palladium(II) [171], silver(I) [172], and paramagnetic nickel(II) [173,174]. Diamagnetic Ag(I) was used as a probe for diamagnetic Cu(I)-binding in a system where Cu(II) and Cu(I) could interchange through redox [180]. Paramagnetic Co(III) was substituted with diamagnetic Ga(III) while studying peptides representing a ferromagnetic Fe(III) binding protein motif, as Ga(III) has the same coordination behavior as Fe(III) [181,182] (Note that gallium should be handled in a glove box). Diamagnetic Ca(II) was substituted by Tb(III) as a paramagnetic probe for long-range intermolecular interactions [183].

## 10. Some Non-Essential Elements

A brief survey of some of the non-essential elements that occur in metal ion-peptide complexes studied by NMR include some that are used to exchange essential ions from experimental considerations and some that are prevalent in the environment and whose effect on biologic systems are studied.

A number of non-essential metals are implicated in playing a role in Alzheimer’s disease and their interactions with amyloid beta-derived peptides were studied to gain insights into possible mechanisms of action, e.g., interactions with aluminum [184] and palladium [171].

*Nickel* NMR measurement can show if a nickel ion binds in diamagnetic form and can therefore serve as a diamagnetic model for paramagnetic ions which would not otherwise be structurally accessible [185]. Studies that compare apo and bound forms of Ni(II) binding peptides provide insight into the role of nickel complexation in structure and allude to the role paramagnetic ions potentially play.

The aforementioned amino-terminal copper- and nickel-binding (ATCUN) motif is found in numerous proteins and its binding properties have been studied using linear and cyclic peptide models with Co(II), Ni(II) and other divalent ions [89,171,186].

Nickel itself is implicated in metal-induced toxicity and carcinogenesis, and Ni(II)-peptide models derived from the C-terminal of histone H2B were studied to elucidate the role this protein may play in carcinogenesis [187]. Nickel can elicit allergic responses for which interactions of Ni(II) with a peptide derived from the human Toll-like receptor (hTLR4) were studied [188].

*Gallium* is diamagnetic and has been shown to substitute Fe(III) binding in Fe_2_S_2_ clusters with a full preservation of structure [189].

*Palladium* has been used as a diamagnetic model for Cu(II) binding in amyloid beta-derived peptides [171] and prion protein peptide derivatives [190] as it has the same square-planar geometry, although it is slightly larger. The dependence of palladium coordination on pH was also studied using Pd(II)-peptide complexes [191]. Peptides were used to study the difference between the binding mode of Pd(II) relative to that of Pt(II) binding, due to interest in the strong anti-tumor propensity of the latter ion together with its toxicity [192].

*Silver* is a toxic diamagnetic ion that binds the human copper transporter 1 (hCtr1). The structure of an Ag(I) complex with a micelle-bound peptide derived from this Cu(I) transporter was derived and, when compared to the free peptide, suggested that the membrane surface may affect the structure of the extracellular domain of the protein and its binding to Ag(I) [193]. The structure of a peptide derived from the human copper transporter 2 (hCtr2), which is a low-affinity homologue of the aforementioned hCtr1, in the presence and absence of Ag(I), showed Ag(I)-binding occurred when the peptide was in trimeric form [172].

*Platinum* is also diamagnetic and is important due to its anticancer properties. Pt(II)-binding of a peptide comprising the receptor binding sequence of transferrin was determined by titration and followed by NMR [194].

*Lanthanides* are almost all paramagnetic and routinely used as shift reagents to follow significant changes in chemical shift that occur in their proximity. Much of the work done with lanthanides and peptides has been to conjugate the lanthanide-peptide complex to a protein to utilize the properties of the shift reagent on the protein. One study gave the NMR-derived structures of fibrinopeptide B, a thrombosis factor, determined in the presence of salts, including Ga(III) [163]. 1D ^1^H-NMR was used to investigate the binding properties of small peptides derived from Ca(II)-binding sites with the toxic La(III), Eu(III) and Tb(III), that have similar ionic radii as Ca(II), to ascertain whether they could be strongly complexed for diagnostic use as contrasting agents [195].

## 11. Considerations in Choosing Metals

In most cases the choice of metal for peptide-binding studies will be dictated by the specificity of the parent protein. However, there may be cases where alternate metals can be considered. Particularly in cases of diamagnetic versus paramagnetic metals, there will often be considerations under which one is preferable to the other. In these cases, the size and geometry of binding will have to be taken under consideration in order to maintain the coordination geometry and a similar binding constant. Since NMR measures the conglomerate motion of the peptide-metal complex over time, the binding constant may have a strong influence over the accuracy and precision of the obtained ensemble. Often the comparison between peptide complexes with different metals will be able to provide deeper insight into the system. Newer methodology is able to take advantage of the fast relaxation of paramagnetic systems and weigh the resulting average structure towards a less prevalent conformation, providing new information on the more transient molecular structure [29,83,196,197].

Other considerations are secondary, however the ease with which a sample can be prepared and measured will ultimately contribute to the success of the experiment: Ions whose oxidation states are unstable will require particular care in handling and usually require an oxygen-free environment during sample preparation and a sealed NMR tube to prevent subsequent degradation. Toxicity and expense of the metals may also factor into the decision of which metal to use.

## 12. Outlook

Over a third of the known proteins bind metal which suggests that a comprehensive understanding of metal-binding in proteins is necessary to understand both their structural stability and their activity. The ability to use solution-state NMR allows us to change the environment in a measured manner in order to mimic the conditions under which biological activity will occur. Peptides add another approach by which we can fully elucidate the specific interactions at the binding site. In many cases it is unnecessary to solve the structure of the entire protein to obtain the information of the interacting functional groups, and peptides can be used as models instead.

There is a huge number of studies that have been done on metal-peptide systems where the binding mode was inferred from many different types of experiments but was not measured directly to provide an experimentally derived structure. Some of these systems could provide additional structural and mechanistic information if the precise mode of binding were to be known. New experimental methods together with metal ion substitution can be used to elucidate the coordination ligands.

Peptides themselves are increasingly being identified as drug leads and drugs, and metal ion binding can be used to increase selectivity and specificity in these systems by preferentially stabilizing bioactive conformations. The fact that peptides are metabolically unstable is considered an inherent difficulty in their use. However, if their lifetime can be extended through stabilization via metal chelation, we can make use of cases where metallopeptides will be completely degraded and together with the resulting soluble metal ion will either be repurposed within the body or excreted, leaving no residue. This can extend the bioavailability and safety profile of currently used peptide drugs.

Metal-protein interactions that are implicated in disease, in addition to heavy metal toxicity, are systems that are still under investigation and their elucidation can potentially be furthered with the means of peptide models. Peptide models for proteins that can bind multiple ions and proteins that are specific for a single metal that can be exchanged by a toxic metal are all potential systems of interest.

Metal-peptide chelates can be used as metallopeptide delivery systems that will protect the metal ion from adverse chemical reactions with the environment, such as oxidation, and potentially solubilize the metal ions to enable improved bioavailability and reduced side-effects. This is relevant for therapeutic peptide complexes as well as nutritional ones [198].

Metallopeptide-based supramolecular structures are increasingly being investigated and new applications are being explored [129,199]. While they are beyond the realm of solution-state NMR, understanding the basic metallopeptide interactions may help shed light on the molecular structures of these systems and the interactions that govern their construction.

As seen in the case of molybdenum, there are accessible systems that NMR has not yet been applied to. Models of these complexes can surely be used to better understand their vital action and promote promising applications.

Although this summary of the most recent empirical advances in the application of NMR to metal-peptide systems is unable to cover all the studies in the field, it hopes to both review the current state of experimentation and be a basis for new ideas and strategies to develop and learn from the vast world that peptide-chelated metals represent.
